# Chronic Energy Restriction Is Associated with Impaired Follicular Development and Hepatic Bile Acid Remodeling in Mice

**DOI:** 10.3390/ani16193025

**Published:** 2026-09-25

**Authors:** Wenjun Zhu, Jiahao Li, Xiaochan Cheng, Hengyu Zhang, Li Li, Hengxi Wei, Shouquan Zhang

**Affiliations:** 1 Henan Provincial Engineering Research Center for Animal Germplasm Resources Exploration and Innovative Utilization, College of Biology and Food, Shangqiu Normal University, Shangqiu 476000, China; m18672564925@163.com; 2 State Key Laboratory of Swine and Poultry Breeding Industry, National Engineering Research Center for Breeding Swine Industry, College of Animal Science, South China Agricultural University, Guangzhou 510642, China; lijiahao0719@yeah.net (J.L.); yan1275768016@163.com (X.C.); 15307185365@163.com (H.Z.); lili007@scau.edu.cn (L.L.); weihengxi@scau.edu.cn (H.W.)

**Keywords:** chronic energy restriction, bile acids, ovarian steroidogenesis, farnesoid X receptor, follicular development

## Abstract

Chronic energy restriction (ER) can impair female reproductive function, but the peripheral metabolic mechanisms by which sustained energy deficiency disrupts follicular development remain poorly understood. This study aimed to determine whether hepatic bile acid (BA) remodeling contributes to the link between systemic energy availability and ovarian dysfunction. A chronic ER mouse model was established by reducing energy intake by 50% for 44 days. Multi-omics approaches, including hepatic transcriptomics, untargeted metabolomics, and targeted BA metabolomics, were integrated with ovarian functional analyses. ER significantly reduced ovarian weight, impaired follicular development, and decreased circulating concentrations of reproductive hormones. Hepatic transcriptomic and metabolomic analyses revealed extensive metabolic remodeling, with marked enrichment of BA-related pathways and increased expression of CYP7A1, suggesting increased hepatic BA synthetic capacity. Targeted BA profiling demonstrated elevated hepatic and circulating BA levels, including increased concentrations of several farnesoid X receptor (FXR)-activating BAs. Furthermore, ovarian FXR expression was increased, accompanied by decreased expression of key steroidogenic regulators, including SRB1, StAR, CYP11A1, and HSD3B1, consistent with reduced ovarian steroidogenic activity. These findings suggest that altered BA metabolism and increased ovarian FXR expression may contribute to reproductive dysfunction under chronic ER.

## Simple Summary

Energy availability is essential for maintaining female reproductive health. Chronic energy restriction (ER), which reduces energy availability relative to physiological requirements, is a common experimental approach to study the reproductive consequences of sustained energy deficiency and is relevant to nutritional challenges associated with reduced fertility in high-producing livestock. However, the mechanisms linking energy deficiency to ovarian dysfunction remain incompletely understood. In this study, we established a chronic ER mouse model and investigated the role of hepatic bile acid metabolism in reproductive impairment. We found that ER altered hepatic bile acid metabolism, increased circulating levels of specific bile acids, and increased ovarian FXR expression. These alterations were accompanied by reduced expression of key steroidogenic genes, including SRB1, StAR, CYP11A1, and HSD3B1, and impaired follicular development. Our findings provide new insights into the potential role of bile acid signaling in reproductive responses to chronic nutritional restriction.

## 1. Introduction

Reproductive efficiency is one of the major determinants of livestock production performance and economic sustainability. Female reproductive performance depends on the coordinated regulation of multiple physiological processes, including nutritional status, endocrine signaling, ovarian follicular development, and embryonic developmental competence. In modern intensive livestock production systems, nutritional imbalance has become a critical factor limiting reproductive efficiency, particularly in high-producing dairy cows and lactating sows [1,2]. Negative energy balance (NEB), defined as a physiological state in which energy expenditure exceeds dietary energy intake, is commonly observed during the early lactation period in dairy cows and is closely associated with delayed resumption of ovarian cyclicity, reduced oocyte quality, and decreased conception rates [3,4]. Similar metabolic challenges occur in sows, especially primiparous lactating sows. Due to high lactation demands and limited feed intake capacity, sows are prone to severe NEB, which subsequently impairs follicular development, oocyte maturation, and subsequent embryonic developmental potential [5].

The reproductive consequences of energy restriction (ER) have traditionally been attributed primarily to disruption of the hypothalamic–pituitary–gonadal (HPG) axis. In dairy cows, NEB is associated with altered circulating insulin and IGF-1 concentrations, as well as reduced LH pulse frequency, thereby delaying follicular development and ovulation [3,6]. However, endocrine alterations alone may not fully explain the detrimental effects of reduced energy availability on ovarian function. Evidence from controlled ER studies further indicates that limiting energy intake can reproduce key reproductive consequences of energy deficiency, as demonstrated by impaired follicular growth and oocyte developmental competence and altered IGF-1 and steroid hormone concentrations within follicular fluid in lactating sows [5]. Similarly, in dairy cows, NEB significantly alters the metabolic composition of the follicular microenvironment, including increased exposure of follicles to non-esterified fatty acids and other metabolic intermediates, thereby negatively affecting granulosa cell function and oocyte developmental competence [7,8]. Therefore, beyond the classical HPG axis, whether sustained reduction in energy availability induces peripheral metabolic remodeling and generates circulating metabolic signals that directly influence ovarian function may represent a critical mechanism linking energy status with reproductive performance.

The liver serves as a central organ responsible for systemic energy metabolism and adaptation to nutritional stress. In response to sustained reductions in energy availability, the liver plays essential roles in fatty acid oxidation, gluconeogenesis, and lipid metabolism. In high-producing dairy cows and lactating sows, persistent energy deficiency induces extensive adipose mobilization and increases hepatic metabolic burden. When these compensatory responses exceed hepatic adaptive capacity, they may lead to lipid accumulation, oxidative stress, and metabolic disorders [9,10]. Notably, recent studies have revealed that NEB-associated lipid metabolic stress may also disrupt hepatic bile acid (BA) homeostasis. Periparturient dairy cows experiencing severe NEB and fatty liver exhibited elevated concentrations of total BAs in both liver and serum, suggesting a close association between energy metabolic stress and hepatic BA dysregulation [11]. Similarly, lactating sows with greater body weight loss exhibit extensive plasma metabolic remodeling, with differentially abundant metabolites enriched in pathways related to fatty acid oxidation and BA biosynthesis [12]. Further studies have shown that enhanced lipid mobilization during lactation in sows is accompanied by substantial alterations in BA metabolism, characterized by an expanded circulating BA pool and remodeling of BA composition, including markedly increased levels of CDCA and LCA [13]. Because BAs can enter the circulation and act on distant tissues, hepatic BA metabolic remodeling may therefore represent a potential metabolic pathway connecting energy deficiency with reproductive organ function.

Beyond their classical role in lipid digestion and absorption, BAs function as important metabolic signaling molecules by activating receptors such as the farnesoid X receptor (FXR) and the G protein-coupled BA receptor 1 (TGR5/GPBAR1), thereby regulating glucose and lipid metabolism, inflammatory responses, and energy homeostasis [14,15]. Increasing evidence suggests that BAs may participate in the regulation of the ovarian microenvironment. In lactating dairy cows, elevated total BA concentrations have been detected in dominant follicular fluid, accompanied by increased expression of the BA transporter SLC10A2 and the receptor GPBAR1 in granulosa cells, suggesting that lactation-associated metabolic stress may enhance local ovarian BA signaling [16]. In buffaloes and pigs, accumulation of specific BAs, such as cholic acid and glycodeoxycholic acid, in atretic follicles is associated with increased granulosa cell apoptosis and impaired steroidogenesis [17,18]. Our previous study further demonstrated that cholic acid suppresses steroidogenesis and follicular development through activation of ovarian FXR signaling in mice [19]. These findings suggest that BA signaling may serve as a mediator linking metabolic status to ovarian function. However, whether ER-induced metabolic remodeling contributes to impaired follicular development remains poorly understood, particularly with respect to the potential involvement of hepatic BA metabolism.

Therefore, the present study aimed to determine whether hepatic BA metabolic remodeling contributes to the impairment of follicular development induced by chronic ER. To experimentally investigate the reproductive consequences of sustained energy deficiency under controlled conditions, we established a chronic ER mouse model through sustained reduction in energy intake and integrated transcriptomic, metabolomic, and targeted BA metabolomic approaches to systematically characterize alterations in hepatic BA metabolism, circulating BA profiles, and ovarian function. We hypothesized that chronic ER induces hepatic BA metabolic remodeling and alters circulating BA composition, thereby increasing ovarian exposure to FXR-activating BAs and subsequently affecting ovarian FXR-related signaling, steroidogenesis, and follicular development. Elucidation of this potential peripheral metabolic pathway will provide new insights into mechanisms linking sustained energy deficiency to female reproductive dysfunction and may help clarify the metabolic basis of reproductive impairment associated with NEB.

## 2. Materials and Methods

### 2.1. Animals and Ethical Approval

Six-month-old female Kunming mice were selected to represent mature adult females and to investigate the effects of chronic ER under physiologically mature conditions. The mice were obtained from the Guangdong Medical Laboratory Animal Center (Foshan, China; License No. SCXK [Yue] 2022-0002). Animals were housed under controlled conditions (12 h light/dark cycle, 22 ± 2 °C, 50–60% humidity) with free access to water and a standard chow diet. The standard chow was obtained from the Guangdong Medical Laboratory Animal Center (Foshan, China). All experimental procedures were approved by the Animal Ethics Committee of South China Agricultural University (Approval No. 2024F005) and conducted in accordance with institutional and national guidelines for the care and use of laboratory animals.

### 2.2. Experimental Design

Following a one-week acclimation period, mice were randomly assigned to the control (CON) or energy restriction (ER) group. Mice in the CON group were provided with a standard chow diet ad libitum, whereas mice in the ER group received 50% of the mean daily food intake of CON mice for 44 consecutive days. Food intake was recorded daily for each mouse in the CON group, and the amount of chow provided to the ER group was adjusted daily based on the mean food intake of CON mice on the preceding day. All mice were individually housed and had free access to water. Food was administered once daily at a fixed time (6:00 p.m.) to minimize variability. Body weight was recorded every 4 days, and body composition was assessed on days 7 and 42.

After the 44-day dietary intervention, estrous cycles were monitored daily for 3 consecutive days using vaginal cytology. The purpose of estrous-cycle monitoring was to standardize the ovarian sampling stage rather than to characterize complete estrous-cycle length or cyclicity. Mice identified as being in diestrus were selected for the subsequent experiments. From the CON group, 22 diestrus mice were selected, with 11 assigned to the main CON group and 11 assigned to the CON+PMSG group. Similarly, 22 diestrus mice were selected from the ER group, with 11 assigned to the main ER group and 11 assigned to the ER+PMSG group. Thus, 11 mice per group were included in each of the four final experimental groups.

Mice in the main CON and ER groups were used for the primary experimental analyses after the 44-day dietary intervention. For selected downstream analyses, eight mice per group were randomly selected for qPCR analyses, whereas four mice per group were randomly selected for Western blot analysis and ovarian follicle quantification. No animals were excluded from the study after group allocation.

Mice in the CON+PMSG and ER+PMSG groups received an intraperitoneal injection of pregnant mare serum gonadotropin (PMSG; 10 IU/mouse; Ningbo Second Hormone Factory [NSHF], Ningbo, China) after confirmation of the diestrus stage, while maintaining their assigned dietary conditions for the following 48 h. Mice were euthanized 48 h after PMSG administration, and ovaries were collected to evaluate ovarian responsiveness to gonadotropin stimulation and follicular development. The corresponding results are presented in the Supplementary Materials.

### 2.3. Sample Collection

Mice were fasted for 12 h before sample collection, with sampling performed the following morning. Following isoflurane anesthesia, blood was collected and allowed to clot at room temperature for 30 min before centrifugation (4000 rpm, 10 min, 4 °C). Serum was stored at −80 °C. Mice were then euthanized by cervical dislocation. Ovaries, liver, and gallbladders were collected, weighed, and processed for downstream analyses. One ovary and the right lobe of the liver were fixed in 4% paraformaldehyde, while remaining tissues were snap-frozen in liquid nitrogen.

### 2.4. Body Composition Analysis

Body composition was assessed using a small-animal quantitative magnetic resonance system (MesoQMR 23-060H-I; Nuimag Corp., Shanghai, China). Prior to scanning, each mouse was weighed. Mice were gently restrained and placed into the instrument’s scanning cylinder. Measurements were performed using the device’s default live-scan protocol. The outputs included percentage fat mass (fat mass/body weight × 100%) and percentage lean mass (lean mass/body weight × 100%).

### 2.5. Biochemical Analysis

Serum levels of alanine aminotransferase (ALT), aspartate aminotransferase (AST), alkaline phosphatase (ALP), total BAs, glucose, and creatinine, as well as hepatic total BAs, were measured using colorimetric assays kits (Elabscience Biotechnology Co., Ltd., Wuhan, China) according to the manufacturers’ instructions. Absorbance was measured at appropriate wavelengths using a spectrophotometer (Thermo Fisher Scientific, Madison, WI, USA).

Serum concentrations of insulin, IGF-1, FSH, LH, progesterone, and estradiol were measured using enzyme-linked immunosorbent assay kits (Elabscience Biotechnology) according to the manufacturers’ protocols. Absorbance was read at 450 nm using a spectrophotometer (Thermo Fisher Scientific). All assays were performed with appropriate standard curves, and intra- and inter-assay coefficients of variation were below 10%.

### 2.6. Histological Analysis

Fixed liver and ovarian tissues were dehydrated, embedded in paraffin, and sectioned by Servicebio (Wuhan, China). Paraffin-embedded blocks were sectioned using a microtome (RM2235, Leica, Wetzlar, Germany). Ovarian tissues were serially sectioned at 7 μm thickness, and every fifth section was mounted onto glass slide until the entire ovary was processed. Liver tissues were sectioned at 10 μm. All sections were stained with hematoxylin and eosin (H&E) and imaged at 20× magnification using a digital research microscope (PreciPoint M8, PreciPoint, Freising, Germany).

Histological evaluation and follicle classification were performed by two investigators blinded to group allocation. Follicle classification and quantification were performed as previously described [19,20,21]. Briefly, preantral follicles were identified as structures with multiple layers of columnar granulosa cells and no visible antrum or only a small cavity smaller than the oocyte. Only morphologically intact follicles with clearly visible oocyte nuclei were included. The counts of preantral follicles were adjusted by a factor of five to account for the sampling frequency. Healthy antral follicles were defined by the presence of an intact oocyte, a fluid-filled antral cavity comparable in size to the oocyte, multiple layers of granulosa cells, and a distinct theca cell layer. Atretic antral follicles were characterized by dispersed granulosa cells, a fragmented zona pellucida, more than 5% of granulosa cells showing morphological features of apoptosis, and a deformed oocyte. This criterion was applied consistently across all serial sections evaluated for atretic follicle classification. The total numbers of healthy and atretic antral follicles, as well as corpora lutea, in each ovary were determined by sequentially tracking individual structures across the serial sections in Adobe Illustrator (Version 27.5.0; Adobe Inc., San Jose, CA, USA) from their first appearance until they were no longer visible, following the approach described in our previous study [19]. Each antral follicle and corpus luteum was counted only once across the serial sections to avoid duplicate counting.

### 2.7. RNA-Seq Analysis of Liver and Ovarian Tissues

RNA-seq library construction and sequencing were performed by LC-Bio Technology Co., Ltd. (Hangzhou, China) following standard protocols [22]. Total RNA was extracted from mouse liver and ovarian tissues using TRIzol reagent (Invitrogen, Carlsbad, CA, USA). RNA quality was assessed with a Bioanalyzer 2100 (Agilent, Santa Clara, CA, USA), and only samples with concentration > 50 ng/μL, RIN > 7.0, and total RNA > 1 μg were used for library preparation. For each tissue type, nine mice per group were randomly assigned to three pools, with tissues from three mice combined to generate each pooled sample. Thus, three pooled biological samples per group were used for RNA-seq library construction and sequencing. mRNA was enriched using oligo magnetic beads (Thermo Fisher Scientific), fragmented, and used for strand-specific library construction with the dUTP method. Libraries (~300 ± 50 bp) were amplified for 8 cycles and sequenced on an Illumina NovaSeq 6000 platform (LC-Bio Technology) in paired-end 150 bp mode. Raw reads were processed with Cutadapt (v1.9) to remove adapters and low-quality sequences. Clean reads were aligned to the mouse reference genome (GRCm38) using HISAT2 (v2.2.1). Transcript assembly and FPKM quantification were performed with StringTie (v2.1.6). Differential expression analysis was performed using gene-level raw read counts with DESeq2 (v1.40.2). Genes with an absolute log_2_ fold change ≥1 and a Benjamini–Hochberg-adjusted *p* value (*q* value) < 0.05 were considered differentially expressed genes (DEGs). GO and KEGG enrichment analyses were performed using the statistically supported DEG sets with DAVID.

### 2.8. Untargeted Metabolomic Analysis of Liver Tissues

Untargeted metabolomic profiling of liver tissues was performed by LC-Bio Technology Co., Ltd. (Hangzhou, China) following standard protocols [23]. Liver samples from nine mice per group were pooled into three biological samples, with liver tissues from three mice combined to generate each pooled sample. Therefore, three pooled biological samples per group were subjected to metabolomic analysis. Metabolites were extracted with 50% methanol, and supernatants were collected for UHPLC-MS analysis. Chromatographic separation was performed using a Vanquish Flex UHPLC system (Thermo Fisher Scientific) equipped with an ACQUITY UPLC T3 column (Waters, Milford, MA, USA). Mass spectrometric data were acquired on a Q-Exactive Orbitrap mass spectrometer (Thermo Fisher Scientific) in both positive and negative ion modes. Raw data were processed using XCMS in R for peak detection, alignment, and retention time correction. Metabolic features were annotated using CAMERA and metaX. For MS1-based annotation, accurate mass matching was performed with a mass tolerance of 10 ppm against public databases, including KEGG and HMDB. For MS/MS-based annotation, spectra were evaluated against the HMDB, KEGG, and LIPIDMAPS databases using an MS1 mass tolerance of 0.01 Da, an MS2 mass tolerance of 0.05 Da, and an identification score cutoff of 75%. Retention time information was recorded for each metabolic feature. A pooled quality control (QC) sample was prepared by mixing equal aliquots of the experimental samples and was analyzed at regular intervals throughout the analytical sequence to monitor technical reproducibility. Features with a QC coefficient of variation (CV) > 30% were excluded from downstream analysis. Metabolic features meeting the exploratory criteria of an absolute log_2_ fold change ≥ 1 and a nominal *p* < 0.05 were selected for exploratory analysis. Exploratory KEGG pathway analysis was subsequently performed using metabolic features.

### 2.9. Targeted BA Metabolomic Analysis of Liver and Serum

Targeted BA profiling in liver and serum was conducted by Personalbio Technology Co., Ltd. (Shanghai, China) using UPLC-MS/MS platforms. Samples from nine mice per group were pooled into three biological samples, with samples from three mice combined to generate each pooled sample. Thus, three pooled samples per group were subjected to targeted BA metabolomic analysis. Metabolites were extracted using methanol/acetonitrile with an internal standard and prepared for UPLC-MS/MS analysis following standard protocols. Chromatographic separation was performed on an ACQUITY UPLC HSS T3 C18 column (Waters), and detection was carried out on a QTRAP 6500+ mass spectrometer (SCIEX, Framingham, MA, USA) in negative ion mode using multiple reaction monitoring. Bile acids were identified by comparison with authentic standards, and quantification was performed using calibration curves. Calibration curves were generated using a 1/x weighting scheme across 13 concentration levels ranging from 0.1 to 1000 ng/mL. Correlation coefficients (r) were ≥0.994 for all analytes, and the LLOQ and ULOQ for each BA are provided in Supplementary File S1. Pooled QC samples were injected approximately every 10 analytical samples to monitor instrumental stability. QC total ion chromatograms showed consistent retention times and peak intensities, and QC samples showed high inter-sample correlation. Most QC CVs were <30%, indicating good analytical repeatability. Differentially abundant BAs were defined based on a combined criterion of variable importance in projection (VIP) >1 from orthogonal partial least squares discriminant analysis and nominal *p* < 0.05. This criterion integrates multivariate and univariate analyses for identifying differential BAs.

### 2.10. Real-Time Quantitative PCR (qPCR) Analysis

Total RNA was isolated from liver and ovarian tissues using RNAiso Plus reagent (Takara Bio Inc., Otsu, Japan) according to the supplier’s protocol. RNA concentrations were determined with a NanoDrop One spectrophotometer (Thermo Fisher Scientific). Reverse transcription was carried out using the All-in-One First-Strand cDNA Synthesis Master Mix (Yugong Biotech, Lianyungang, China). Quantitative real-time PCR was conducted using the 2× PolarSignal^®^ SYBR Green Taq Mix (Mikxlife, Guangzhou, China) on a qTOWER3 Real-Time PCR System (Analytik Jena, Jena, Germany), following the manufacturer’s recommendations. Primer sequences (listed in Table A1) were designed via the PrimerBank database (https://pga.mgh.harvard.edu/primerbank/, accessed on 26 April 2024). The relative expression levels of target genes were calculated using the 2^−ΔΔCT^ method, with *β-actin* serving as the internal control.

### 2.11. Western Blot Analysis

Total proteins were extracted from liver and ovarian tissues using a protein extraction kit (KeyGEN Biotech, Nanjing, China) in accordance with the manufacturer’s instructions. Equal amounts of protein were separated via SDS-PAGE and transferred onto PVDF membranes (Immobilon^®^-P, Millipore, Cork, Ireland). Membranes were blocked for 1 h at room temperature in 5% non-fat milk (diluted in TBS with 0.1% Tween-20), followed by incubation with specific primary antibodies either overnight at 4 °C or for 2 h at room temperature. Details of the primary antibodies and dilution ratios are provided in Table A2. After three washes with TBST, membranes were incubated with Alpaca-derived anti-rabbit IgG secondary antibody (1:50,000; Huabio, Hangzhou, China) for 1 h. Protein signals were visualized using the Tanon 5200 Chemiluminescence Imaging System (Tanon, Shanghai, China). Densitometric analysis was performed using ImageJ software (version 1.53k; NIH, Bethesda, MD, USA), and signal intensities were normalized to the respective internal controls.

### 2.12. Statistical Analysis

Data are presented as mean ± standard error of the mean (SEM). Each mouse was considered an independent experimental unit, whereas each pooled biological sample was considered the experimental unit for transcriptomic and metabolomic analyses. Pooled omics data were analyzed separately from individual-animal data. Longitudinal body weight and body composition were analyzed using two-way repeated-measures ANOVA, with Bonferroni correction for between-group comparisons at individual time points. Other comparisons were performed using unpaired two-tailed Student’s *t*-tests. Pearson correlation was used for correlation analyses. Analyses were conducted with IBM SPSS Statistics software (version 26.0; IBM Corp., Armonk, NY, USA). A *p*-value < 0.05 was considered statistically significant.

## 3. Results

### 3.1. ER Alters Body Composition, Systemic Metabolic, and Reproductive Endocrine Parameters

To investigate the effect of chronic ER on female reproductive function, a chronic ER mouse model was established by reducing daily energy intake by 50% for 44 days. Body weight was significantly lower in ER mice from day 8 onward (Figure 1A, Bonferroni-adjusted *p* < 0.01). Body composition analysis further revealed that the ER group exhibited a significant increase in lean mass percentage and a decrease in fat mass percentage at both 1 and 6 weeks (Figure 1B,C, Bonferroni-adjusted *p* < 0.05). Consistent with these changes, serum glucose levels were significantly lower, whereas creatinine levels were significantly higher in the ER group than in controls (Figure 1D,E, *p* < 0.001). Endocrine analysis showed that ER mice had significantly lower circulating levels of insulin, FSH, LH, progesterone, and estradiol (Figure 1F,H–K, *p* < 0.05), whereas IGF1 levels remained unchanged (Figure 1G).

### 3.2. ER Induces Ovarian Atrophy and Impairs Follicular Development

Ovarian weights and ovarian weight indices were both significantly decreased in the ER group (Figure 2A,B, *p* < 0.01). Histological analysis revealed clear structural differences between the two groups (Figure 2C). Quantitative follicle counting demonstrated that the numbers of preantral follicles, healthy antral follicles, atretic antral follicles, total antral follicles, and corpora lutea were all significantly reduced in the ER group compared with the CON group (Figure 2D–H, *p* < 0.05).

To further assess ovarian responsiveness to exogenous gonadotropin stimulation, mice were stimulated with PMSG for 48 h. However, PMSG stimulation failed to fully restore ovarian phenotype in the ER group, as evidenced by significantly lower ovarian weight and ovarian weight indices compared with the CON+PMSG group (Figure A1A,B, *p* < 0.001). In addition, the numbers of healthy antral follicles, total antral follicles, and corpora lutea remained significantly reduced in the ER+PMSG group compared with the CON+PMSG group (*p* < 0.05), whereas no significant differences were observed in preantral follicles or atretic follicles (Figure A1C–H).

### 3.3. ER Is Associated with Hepatic Metabolic Remodeling Involving BA Metabolism

To explore whether hepatic metabolic changes contribute to ovarian dysfunction, liver transcriptomic analysis was performed. A total of 1085 DEGs were identified (Figure 3A,B, Supplementary File S2), which were mainly enriched in pathways related to glucose metabolism, lipid metabolism, and bile secretion (Figure 3C,D). Untargeted metabolomic profiling identified 278 metabolic features meeting the predefined exploratory criteria (Figure 3E,F, Supplementary File S3). Exploratory KEGG pathway analysis of these metabolic features suggested alterations in pathways related to primary BA biosynthesis, secondary BA biosynthesis, and bile secretion (Figure 3G). In addition, both mRNA and protein levels of CYP7A1 in the liver were significantly upregulated in the ER group (Figure 3H–J, *p* < 0.01).

### 3.4. ER Is Associated with Increased Hepatic and Circulating BA Levels and Hepatic Injury

To further characterize the alterations in BA homeostasis associated with chronic ER, targeted BA profiling was performed in both serum and liver. Targeted BA metabolomics revealed distinct clustering between groups in PCA analysis (Figure 4A). Among the 52 detected hepatic BAs, 16 were differentially abundant, of which 13 were significantly increased in the ER group (Figure 4B; Table A3). Total hepatic BA levels were significantly increased in the ER group (Figure 4C, *p* < 0.001). Mice in the ER group also exhibited significantly increased gallbladder weight and gallbladder weight indices (Figure 4D–F, *p* < 0.05). Histological analysis revealed hepatic injury in the ER group, characterized by ballooning degeneration and eosinophilic changes (Figure 4G), which was further supported by significantly elevated serum liver injury markers ALT, AST, and ALP levels (Figure 4H–J, *p* < 0.01). Serum BA profiling demonstrated separation between groups in PCA analysis (Figure 4K). Among the 42 detected serum BAs, 21 were differentially abundant, all of which were significantly increased in the ER group (Figure 4L; Table A4). Notably, several FXR-activating BAs, including CDCA, DCA, LCA, and CA, were markedly increased (Figure 4M; Table A4, *p* < 0.05). Total serum BA levels were 15.5-fold higher in the ER group than in the CON group (Figure 4N, *p* < 0.001). Correlation analysis showed a significant negative correlation between total serum BA levels and ovarian weight (Figure 4O, *r* = –0.694, *p* < 0.001).

### 3.5. ER Is Associated with Alterations in Ovarian Cholesterol and Steroid Metabolism

Given the marked elevation of circulating BAs and their significant negative correlation with ovarian weight, ovarian transcriptomic analysis was further performed. A total of 600 DEGs were identified, including 367 upregulated and 233 downregulated genes (Figure 5A,B, Supplementary File S4). GO enrichment analysis revealed that DEGs were significantly enriched in the cholesterol metabolic process, the steroid metabolic process, the estrogen metabolic process, and cholesterol homeostasis (Figure 5C). KEGG pathway analysis further confirmed enrichment in cholesterol metabolism, steroid hormone biosynthesis, Retinol metabolism, and the PPAR signaling pathway (Figure 5D).

### 3.6. ER Increases Ovarian FXR Expression and Reduces Steroidogenic Regulators Expression

Given the marked elevation of circulating FXR-activating BAs in the ER group, ovarian FXR expression was further evaluated. Ovarian FXR expression was significantly increased at both mRNA and protein levels in the ER group (Figure 6A–C, *p* < 0.01). qPCR validation showed that the mRNA expression levels of *Lhcgr* and *Ldlr* were significantly reduced in the ER group (*p* < 0.05), whereas *Fshr* expression was not significantly altered (Figure 6D,E). More importantly, the mRNA and protein expression levels of key steroidogenic regulators—including SRB1, StAR, CYP11A1, and HSD3B1—were significantly decreased in the ER group (Figure 6F–K, *p* < 0.05). In contrast, *Cyp19a1* mRNA expression was not significantly changed, whereas CYP19A1 protein abundance was significantly reduced in the ER group (Figure 6I–K, *p* < 0.01).

## 4. Discussion

The present study found that chronic ER in adult female mice was associated with reduced ovarian weight, impaired follicular development, decreased ovarian steroidogenic activity, and substantial alterations in hepatic BA metabolism and circulating BA profiles. Sustained 50% ER was accompanied by increased circulating levels of FXR-activating BAs, including CDCA, DCA, LCA, and CA, increased ovarian FXR expression, and reduced expression of key steroidogenic genes, including SRB1, StAR, CYP11A1, and HSD3B1. Taken together, these findings support a potential BA–FXR mechanism that may contribute to ovarian dysfunction under chronic ER conditions, providing a possible peripheral metabolic link between sustained energy deficiency and impaired ovarian function.

In the present study, 44 days of chronic ER resulted in reduced body weight and fat mass, decreased circulating concentrations of FSH, LH, estradiol, and progesterone, and marked reductions in ovarian weight and the numbers of preantral follicles, healthy antral follicles, total antral follicles, and corpora lutea. Although this experimental ER model does not fully recapitulate production-associated NEB, the observed reproductive alterations resemble several features reported in cattle and pigs experiencing NEB [5,24,25]. Interestingly, circulating IGF-1 concentrations were not significantly altered by chronic ER despite marked changes in body composition and reproductive hormones. This may reflect the adaptive regulation of the IGF-1 axis under chronic energy restriction, which can be influenced by the severity and duration of energy restriction and nutritional status [26].

Traditionally, energy deficiency-induced reproductive suppression has been primarily attributed to central inhibition of the HPG axis. Insufficient energy availability suppresses GnRH pulse secretion, leading to reduced pituitary FSH and LH secretion and consequently impairing follicular recruitment and steroidogenesis [27]. However, exogenous PMSG stimulation after termination of ER treatment failed to fully restore ovarian weight or healthy antral follicle numbers in ER mice. This incomplete response suggests that the ovarian alterations induced by chronic ER may persist despite exogenous gonadotropic stimulation. The reduced expression of *Lhcgr* observed in ER ovaries may partly contribute to the attenuated response to PMSG, while changes in ovarian steroidogenic capacity and metabolic state may also be involved. Together with the unchanged circulating IGF-1 concentrations, these findings suggest that the ovarian response to chronic ER may involve metabolic signals beyond gonadotropic regulation. Indeed, nutritional and metabolic factors, including insulin, non-esterified fatty acids, and nutrient availability, have been implicated in the regulation of ovarian follicular development [28,29,30].

The liver is a central organ coordinating systemic metabolic adaptation to ER. In this study, hepatic transcriptomic and exploratory untargeted metabolomic analysis revealed alterations in pathways related to BA metabolism, accompanied by increased expression of CYP7A1, a rate-limiting enzyme in the classical BA biosynthetic pathway. These findings suggest increased hepatic BA synthetic capacity. Targeted BA profiling further confirmed elevated total BA concentrations in both liver and serum, together with marked accumulation of several FXR-activating BAs. Previous rodent studies have demonstrated that ER enhances enterohepatic BA cycling and alters BA pool composition [31,32]. In dairy cows, lower body condition scores are associated with increased hepatic CYP7A1 mRNA expression and elevated concentrations of taurine-conjugated BAs, suggesting that BA synthesis and transformation participate in metabolic adaptation to energy status [33]. These findings are consistent with altered hepatic BA metabolic capacity and BA accumulation in the present ER model. Furthermore, transition-period dairy cows experiencing severe NEB exhibit increased total BA concentrations in both liver and serum, accompanied by elevated hepatic injury markers, including ALT, AST, and GLDH [11]. Similarly, ER mice in the present study exhibited BA accumulation; gallbladder enlargement; elevated serum ALT, AST, and ALP levels; and hepatic histological damage. These findings suggest that BA metabolic remodeling may initially represent an adaptive response to ER; however, sustained BA accumulation may challenge the capacity to maintain BA homeostasis and may contribute to hepatic injury [34].

Beyond their classical roles in lipid digestion and absorption, BAs function as metabolic signaling molecules that regulate peripheral tissue functions through nuclear receptors such as FXR and membrane receptors such as TGR5 [14,15]. Studies in dairy cows have demonstrated the presence of multiple BA species in follicular fluid, with follicular BA composition showing high similarity to circulating BA profiles. Moreover, increased BA concentrations have been detected in dominant follicular fluid and plasma of lactating dairy cows, suggesting that systemic metabolic status may influence the ovarian BA microenvironment [16,35]. However, excessive BA exposure may exert detrimental effects. In buffaloes, accumulation of glycodeoxycholic acid in atretic follicles promotes granulosa cell apoptosis and suppresses steroid hormone secretion [18]. In the present study, circulating total BA concentrations were inversely associated with ovarian weight, suggesting a potential relationship between systemic BA accumulation and ovarian atrophy under chronic ER conditions.

FXR is a major intracellular receptor for BAs and is expressed in ovarian granulosa cells, where it has been implicated in the regulation of steroidogenic programs. Previous studies have shown that FXR is primarily localized in granulosa cells of secondary and tertiary follicles. Activation of FXR has been reported to induce expression of its downstream target gene SHP (small heterodimer partner), while SHP overexpression suppresses the expression of StAR, CYP11A1, and HSD3B, suggesting a potential role of FXR–SHP signaling in the regulation of steroidogenesis [36]. A similar mechanism has been reported in testicular Leydig cells, where FXR activation induces SHP and DAX-1 (dosage-sensitive sex reversal, adrenal hypoplasia critical region, on chromosome X, gene-1) expression and is associated with reduced transcription of *Cyp11a1*, *Star*, and *Hsd3b1* and reduces testosterone synthesis. Moreover, mice exposed to elevated BA levels during puberty exhibit increased germ cell apoptosis and reduced fertility [37,38]. In the present study, ER mice exhibited a similar pattern of alterations, including increased circulating FXR-activating BAs, elevated ovarian FXR protein expression, and coordinated downregulation of SRB1, StAR, CYP11A1, and HSD3B1. These genes are involved in cholesterol uptake, mitochondrial cholesterol transport, and rate-limiting steps in steroidogenesis, respectively [39,40]. Their reduced expression is consistent with decreased estradiol and progesterone levels and impaired follicular development under chronic ER conditions. Our previous work further demonstrated that exogenous cholic acid suppressed steroidogenic gene expression and impaired follicular development in association with ovarian FXR activation [19]. Together with the circulating BA alterations and increased ovarian FXR expression observed in the present study, these findings support a potential BA–FXR mechanism linking prolonged ER with impaired ovarian steroidogenesis. This observation is also consistent with findings in buffalo atretic follicles, where accumulation of conjugated BAs is accompanied by reduced steroid secretion [18], suggesting a potentially conserved relationship between BA signaling and gonadal steroidogenesis, which warrants further investigation in production animals such as cattle and pigs.

Several limitations should be considered when interpreting the present findings. The 50% ER model in non-lactating mice does not fully recapitulate production-associated NEB in lactating livestock, and restriction of standard chow simultaneously reduces the intake of other nutrients. For omics analyses, pooling tissues from nine mice into three biological samples per group limited assessment of inter-individual variability, while the 12 h fasting protocol may have influenced circulating BA and metabolic profiles. In addition, the present study did not directly measure ovarian BAs or establish the downstream causal role of FXR, and the observed liver injury may have contributed to the altered BA profiles. These factors should be considered when interpreting the proposed BA–FXR relationship and its relevance to livestock.

## 5. Conclusions

In summary, this study expands our current understanding of how chronic ER impairs female reproductive function in mice and provides potential mechanistic insights relevant to livestock. Long-term ER does not act solely through central inhibition of the HPG axis but is also accompanied by hepatic BA metabolic remodeling, increased circulating levels of FXR-activating BAs, increased ovarian FXR expression, and suppression of key steroidogenic genes. Together, these findings support a potential BA–FXR mechanism linking sustained energy deficiency with impaired ovarian function (Figure 7). Future studies should evaluate BA-lowering nutritional additives, pharmacological modulation of FXR signaling, and bovine and porcine primary ovarian cell models to further investigate the relevance and translational potential of this pathway in livestock.

## Figures and Tables

**Figure 1:**
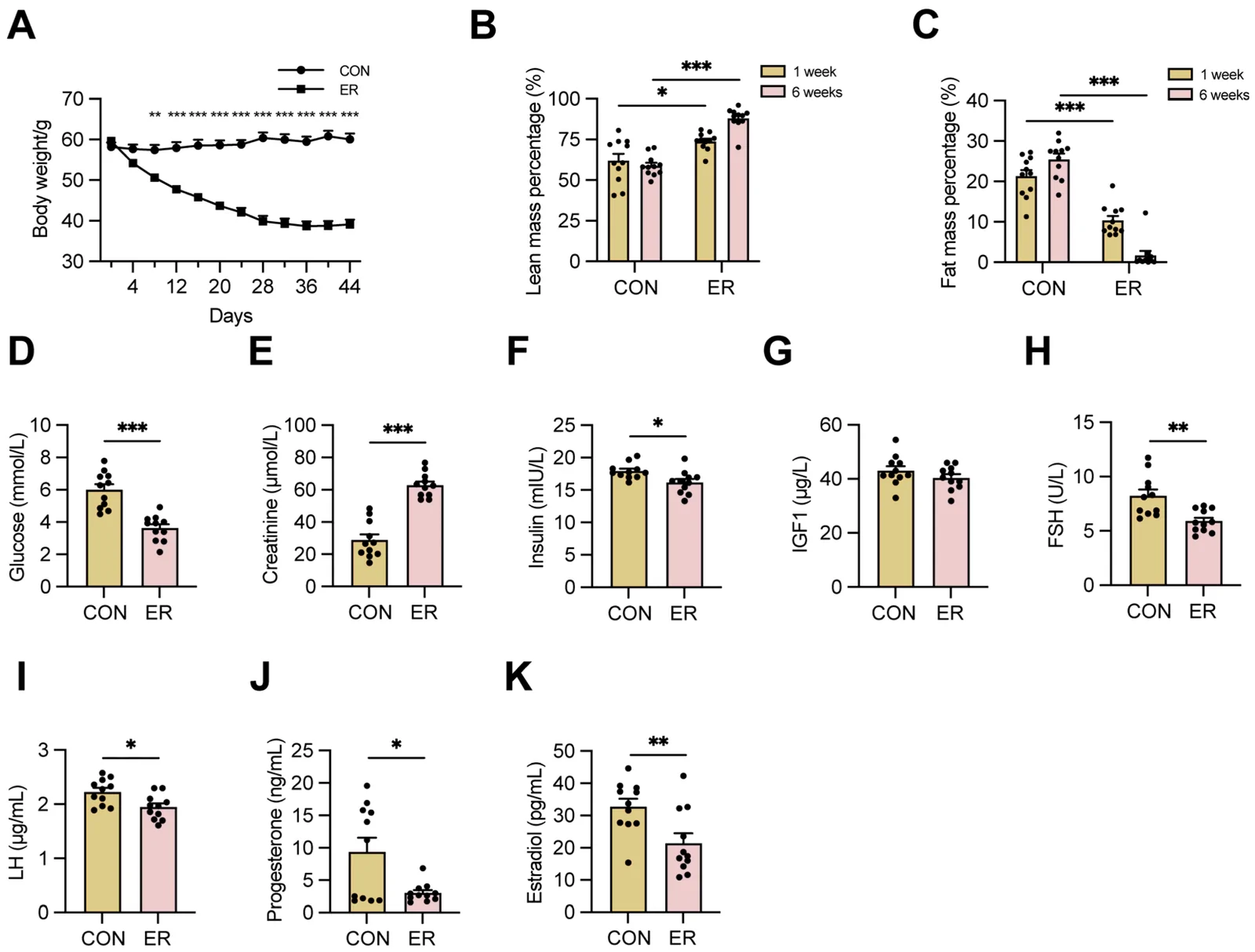
Effects of chronic ER on body weight, body composition, and serum metabolic and endocrine parameters in mice (*n* = 11). (**A**) Body weight changes during the 44-day intervention. (**B**,**C**) Lean mass percentage and fat mass percentage measured on day 7 and day 42. (**D**,**E**) Serum glucose and creatinine concentrations. (**F**–**K**) Serum concentrations of insulin, IGF1, FSH, LH, progesterone, and estradiol. Data are presented as mean ± SEM. Longitudinal data were analyzed using two-way repeated-measures ANOVA, with Bonferroni correction for between-group comparisons at individual time points. Other comparisons were performed using an unpaired two-tailed Student *t*-test. * *p* < 0.05, ** *p* < 0.01, *** *p* < 0.001.

**Figure 2:**
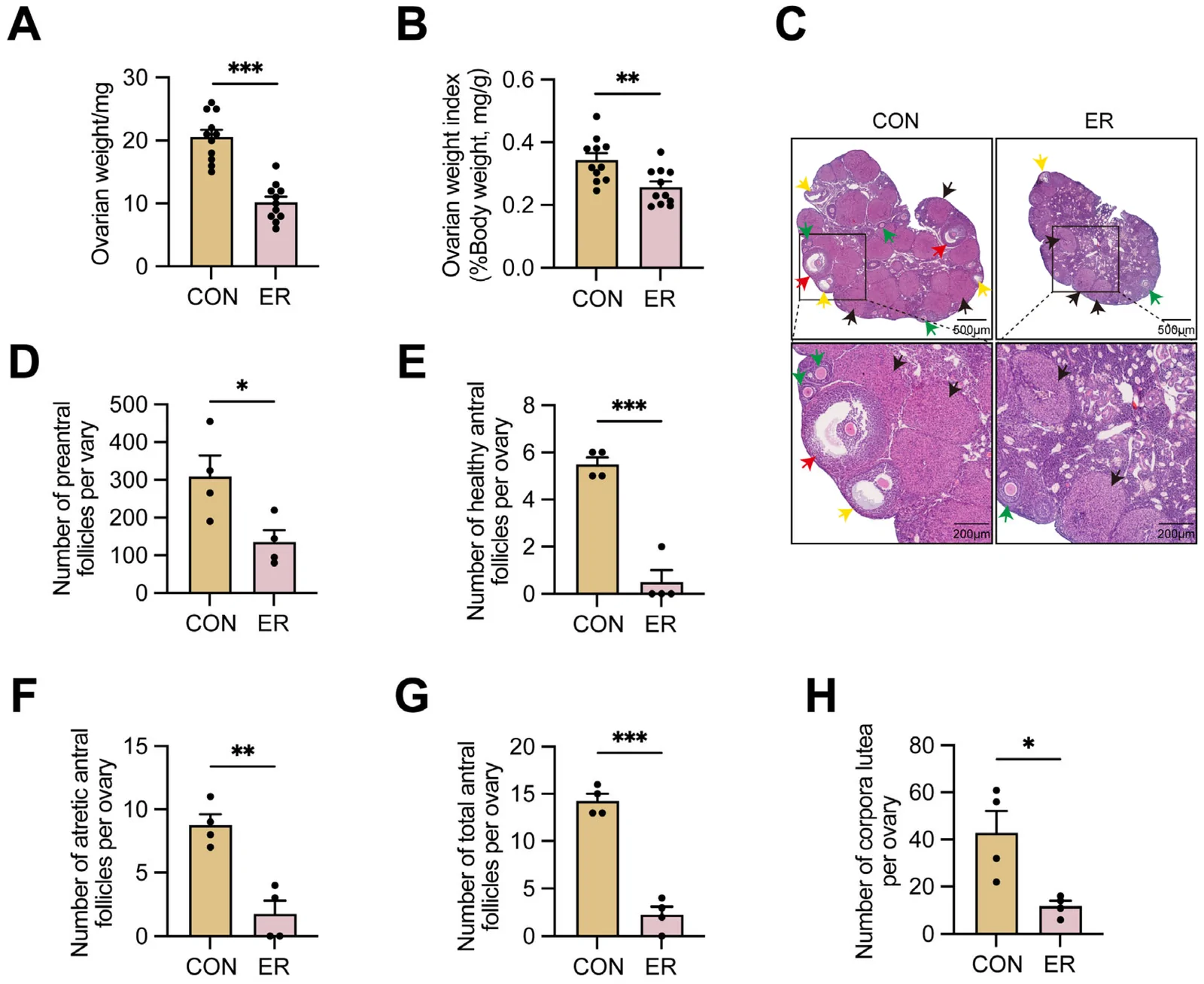
Effects of chronic ER on ovarian weight and follicular development in mice. (**A**,**B**) Ovarian weights and ovarian weight indices (*n* = 11). (**C**) Representative H&E-stained ovarian sections. The green arrow points to the preantral follicles, the red arrow points to the healthy antral follicles, the yellow arrow points to the atretic antral follicles, and the black arrow points to the corpora lutea. Scale bars: 500 μm (upper panel) and 200 μm (lower panel). (**D**–**H**) Quantification of preantral follicles, healthy antral follicles, atretic antral follicles, total antral follicles, and corpora lutea per ovary (*n* = 4). Data are presented as mean ± SEM. Significance was calculated using an unpaired two-tailed Student *t*-test. * *p* < 0.05, ** *p* < 0.01, *** *p* < 0.001.

**Figure 3:**
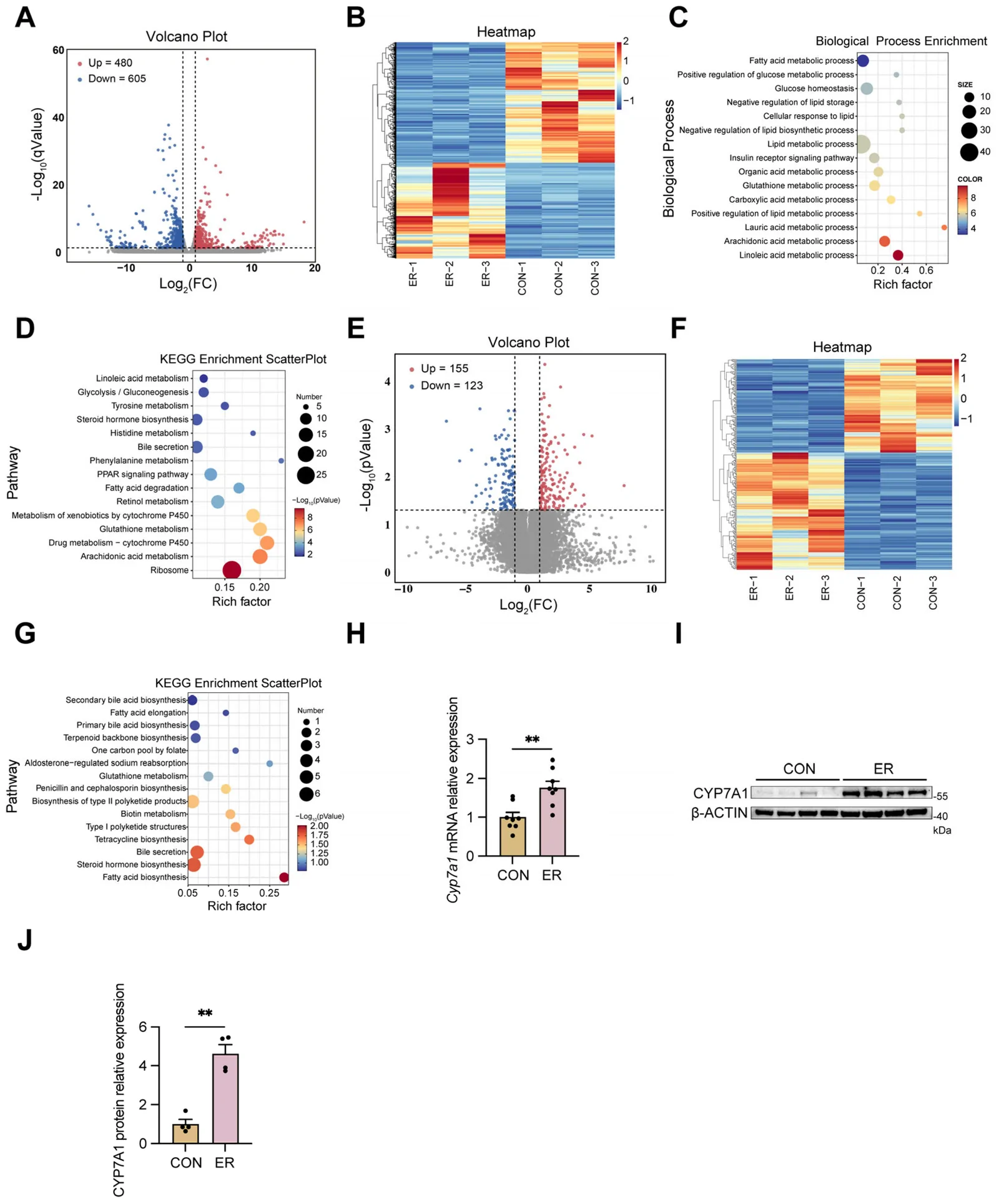
Effects of chronic ER on hepatic transcriptomic and metabolomic profiles in mice. (**A**,**B**) Volcano plot and heatmap showing DEGs between the CON and ER groups identified by RNA-seq. (**C**) GO enrichment analysis of DEGs in biological processes. (**D**) KEGG pathway enrichment analysis of DEGs. (**E**,**F**) Volcano plot and heatmap showing metabolites meeting the predefined exploratory criteria in the liver by untargeted metabolomics. (**G**) Exploratory KEGG pathway analysis of metabolites meeting the nominal criteria. (**H**–**J**) mRNA and protein expression levels of CYP7A1 in the liver. For RNA-seq and untargeted metabolomics, three pooled biological samples per group were analyzed, with each pooled sample generated from tissues of three mice. qPCR and Western blot analyses were performed using eight and four independent mice per group, respectively. Data for qPCR and Western blot analyses are presented as mean ± SEM and were analyzed using an unpaired two-tailed Student *t*-test. ** *p* < 0.01.

**Figure 4:**
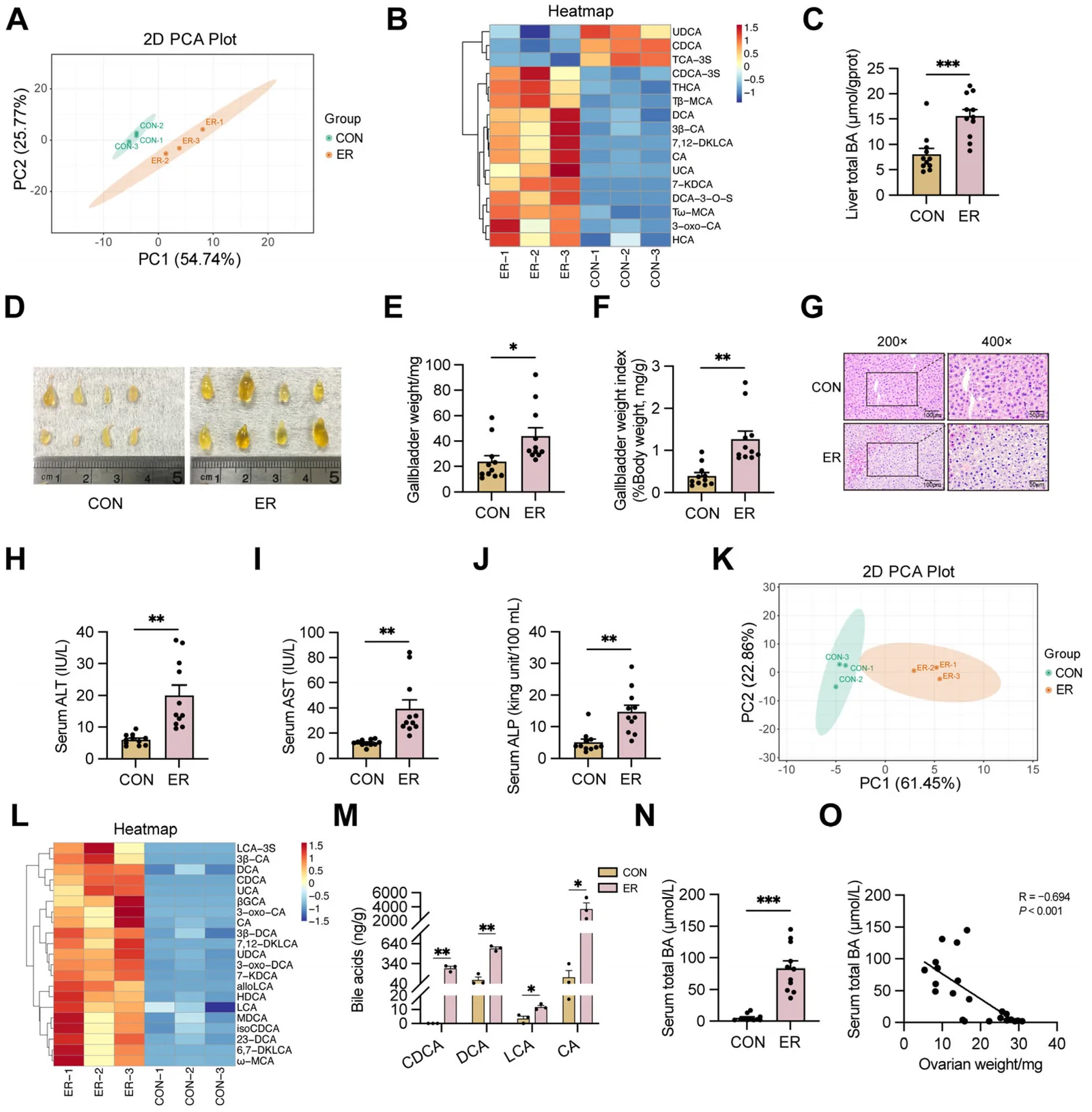
Effects of chronic ER on hepatic and circulating BA profiles and liver injury in mice. (**A**) PCA plot of hepatic BA profiles in the CON and ER groups (*n* = 3). (**B**) Heatmap displaying differentially abundant BAs identified between the CON and ER groups (*n* = 3). (**C**) Total hepatic BA concentrations (*n* = 11). (**D**) Representative images of gallbladders from CON and ER groups. (**E**,**F**) Gallbladder weights and gallbladder weight indices (*n* = 11). (**G**) Representative H&E-stained liver sections. Scale bars: 100 μm (left panel) and 50 μm (right panel). (**H**–**J**) Serum ALT, AST, and ALP activities (*n* = 11). (**K**) PCA plot of serum BA profiles (*n* = 3). (**L**) Heatmap of differentially abundant BAs (*n* = 3). (**M**) Serum concentrations of CDCA, DCA, LCA, and CA measured by targeted metabolomics (*n* = 3). (**N**) Total serum BA levels measured by biochemical assay (*n* = 11). (**O**) Correlation analysis between serum total BA concentration and ovarian weight in each of the 22 individual mice (*n* = 22). For targeted BA metabolomics, three pooled biological samples per group were analyzed, with each pooled sample generated from three mice. Data for individual-animal measurements are presented as mean ± SEM and were analyzed using an unpaired two-tailed Student *t*-test. Pearson correlation analysis was used for panel O. * *p* < 0.05, ** *p* < 0.01, *** *p* < 0.001.

**Figure 5:**
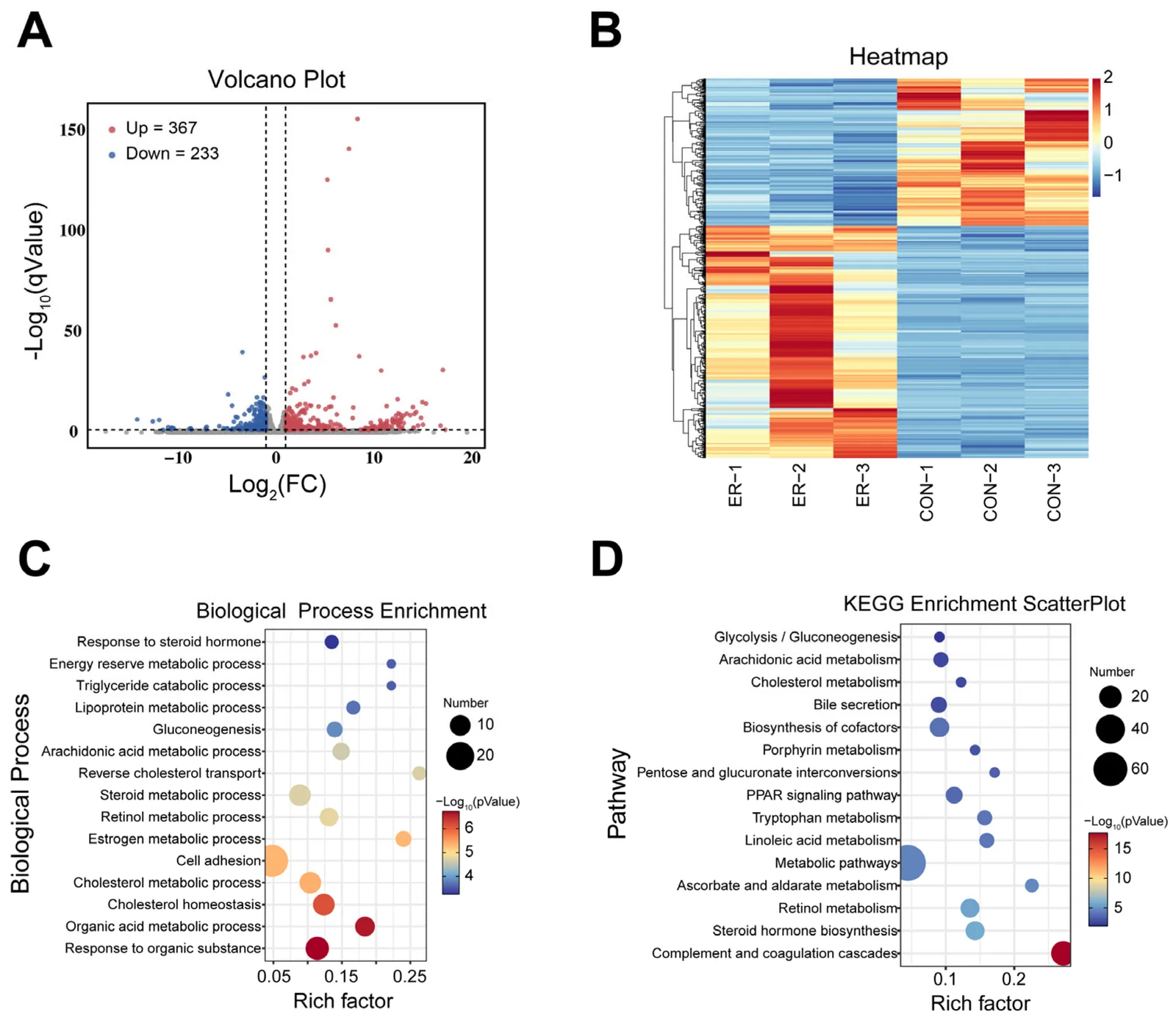
Effects of chronic ER on ovarian transcriptomic profiles in mice. (**A**,**B**) Volcano plot and heatmap showing DEGs between the CON and ER groups identified by RNA-seq. (**C**) GO enrichment analysis of DEGs in biological processes. (**D**) KEGG pathway enrichment analysis of DEGs. Three pooled biological samples per group were analyzed by RNA-seq, with each pooled sample generated from tissues of three mice.

**Figure 6:**
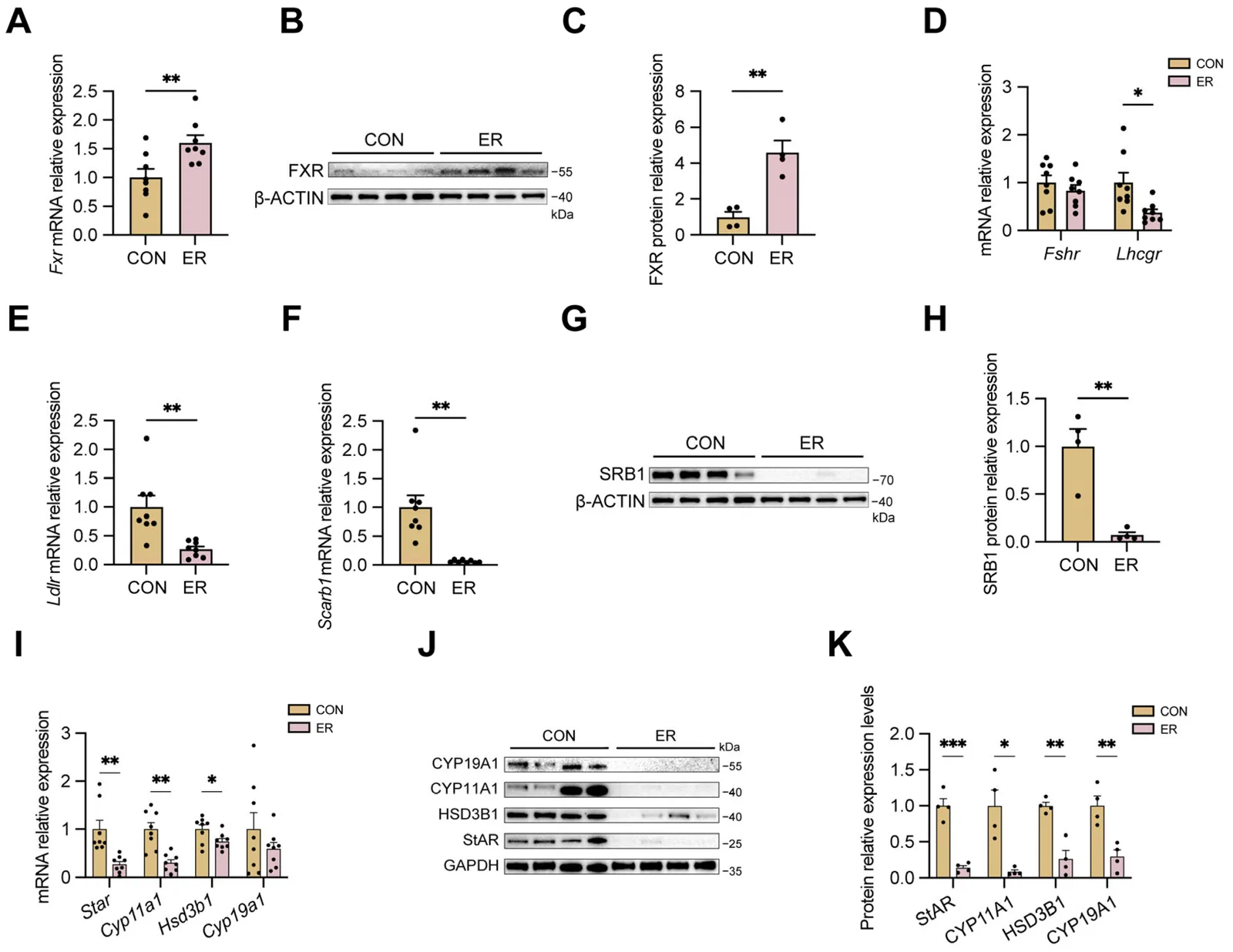
Effects of chronic ER on ovarian FXR expression and steroidogenic regulators. (**A**–**C**) mRNA and protein expression levels of FXR in ovaries. (**D**,**E**) mRNA expression levels of *Fshr*, *Lhcgr*, and *Ldlr*. (**F**–**K**) mRNA and protein levels of SRB1, StAR, CYP11A1, HSD3B1, and CYP19A1. qPCR and Western blot analyses were performed using eight and four independent mice per group, respectively. Data are presented as mean ± SEM. Significance was calculated using an unpaired two-tailed Student *t*-test. * *p* < 0.05, ** *p* < 0.01, *** *p* < 0.001.

**Figure 7:**
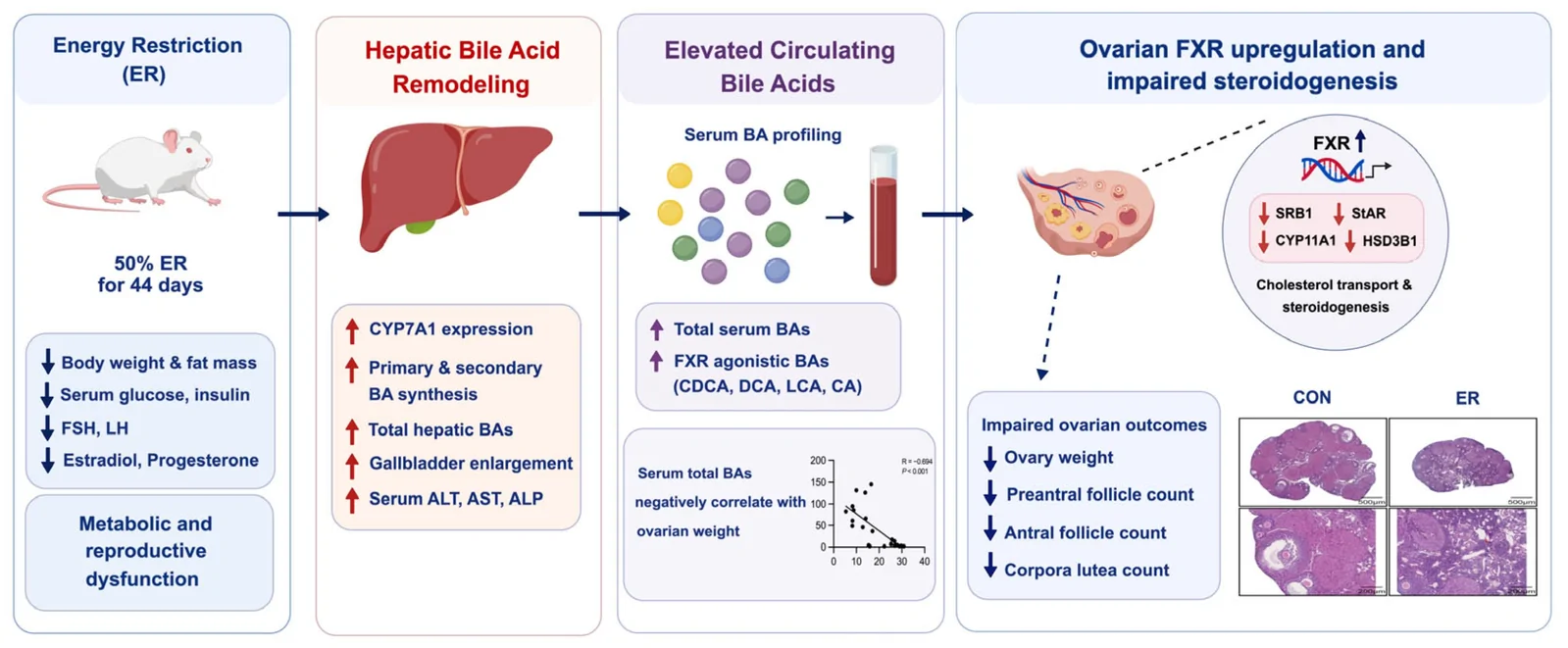
Graphical summary of chronic ER-associated hepatic BA remodeling, elevated circulating BAs levels, and impaired ovarian steroidogenesis. The figure was created with BioGDP.com.

**Figure A1:**
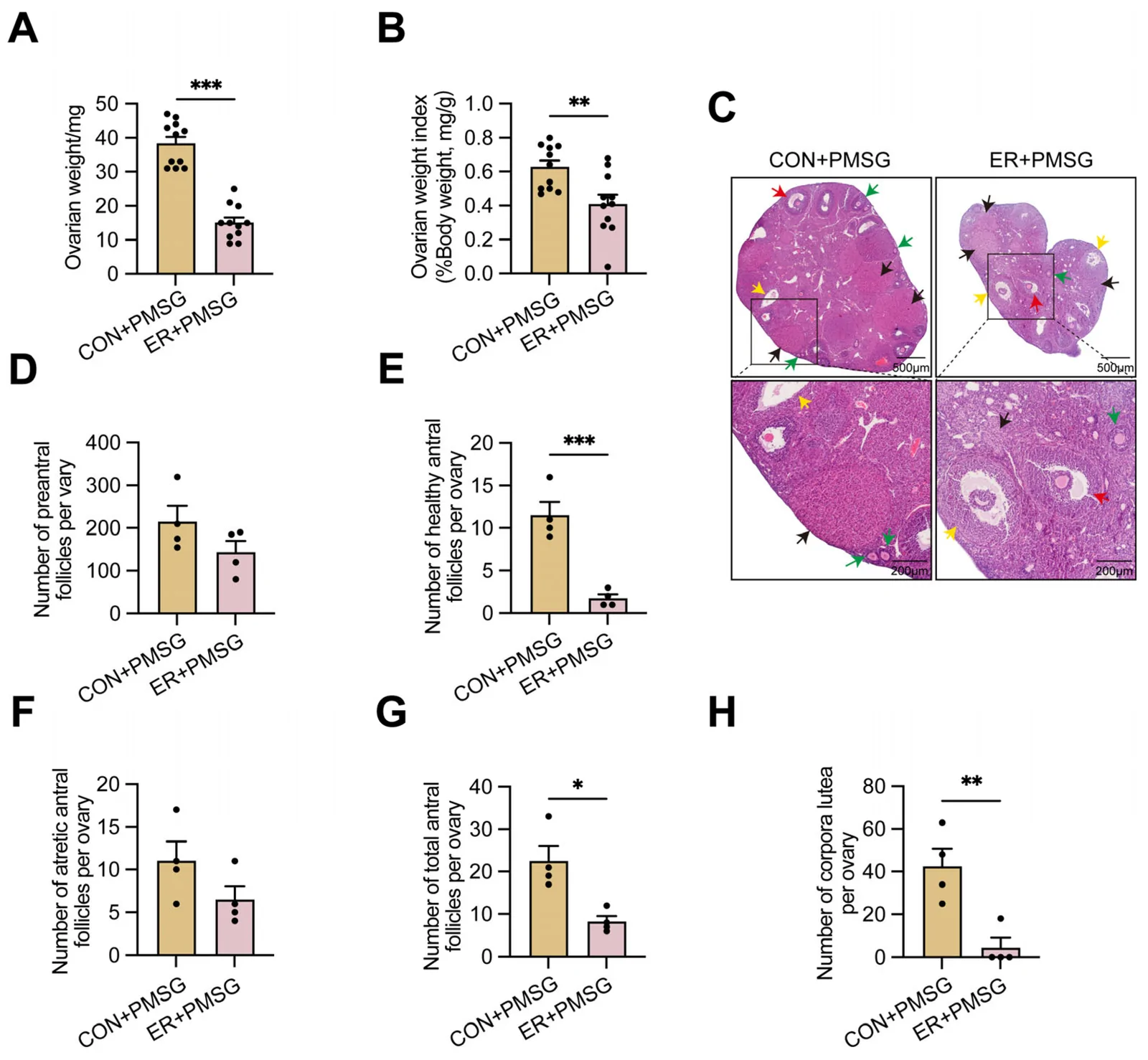
Effect of chronic ER on ovarian follicle development in mice 48 h after PMSG injection. (**A**,**B**) Ovarian weights and ovarian weight indices (*n* = 11). (**C**) Representative images of ovarian histology (H&E staining). The green arrow points to the preantral follicles, the red arrow points to the healthy antral follicles, the yellow arrow points to the atretic antral follicles, and the black arrow points to the corpora lutea; scale bars: upper panel, 500 μm; lower panel, 200 μm. (**D**–**H**) Quantification of preantral follicles, healthy antral follicles, atretic antral follicles, total antral follicles, and corpora lutea per ovary (*n* = 4). Data are presented as mean ± SEM. Significance was calculated using an unpaired two-tailed Student *t*-test. * *p* < 0.05, ** *p* < 0.01, *** *p* < 0.001.

## Data Availability

The original contributions presented in this study are included in the article/Supplementary Material. Further inquiries can be directed to the corresponding author.

## Supplementary Materials

The following supporting information can be downloaded at https://www.mdpi.com/article/10.3390/ani16193025/s1. Files S1: Targeted bile acid metabolomic analysis parameters for mouse liver and serum samples; Files S2: List of differentially expressed genes in liver; Files S3: List of exploratory metabolic features in liver; Files S4: List of differentially expressed genes in ovary.

## Abbreviations

The following abbreviations are used in this manuscript:

ER	Energy Restriction
NEB	Negative energy balance
BA	Bile acid
HPG	Hypothalamic–pituitary–gonadal
FXR	Farnesoid X receptor
TGR5/GPBAR1	G protein-coupled BA receptor 1
CON	Control group
PMSG	Pregnant mare serum gonadotropin
ALT	Alanine aminotransferase
AST	Aspartate aminotransferase
ALP	Alkaline phosphatase
H&E	Hematoxylin and eosin
DEG	Differentially expressed gene
qPCR	Real-time quantitative PCR
SEM	Standard error of the mean
SHP	Small heterodimer partner
DAX-1	Dosage-sensitive sex reversal, adrenal hypoplasia critical region, on chromosome X, gene-1

## Appendix A

**Table A1:** List of Primers Used for qPCR Analysis.

Genes	Primer Sequence (5′-3′)	Product Length (bp)
*Fshr*	F: CCAGCCTTACCTACCCCAGT	76
	R: CAAATTGGATGAAGTTCAGAGGT	
*Lhcgr*	F: GACCAAAAGCTGAGGCTGAGA	78
	R: CAATGTGGCCATCAGGGTAGA	
*Scarb1*	F: TGTACTGCCTAACATCTTGGTCC	126
	R: ACTGTGCGGTTCATAAAAGCA	
*Ldlr*	F: TGACTCAGACGAACAAGGCTG	118
	R: ATCTAGGCAATCTCGGTCTCC	
*Cyp11a1*	F: ACATGGCCAAGATGGTACAGTTG	119
	R: ACGAAGCACCAGGTCATTCAC	
*Star*	F: GTGAAGGCTAAGGGATAA	125
	R: TGGAGCTGGTAAGACAAC	
*Hsd3b1*	F: TGGACAAAGTATTCCGACCAGA	250
	R: GGCACACTTGCTTGAACACAG	
*Cyp19a1*	F: CCCGAGCCTTTGGAGAACAA	161
	R: TGAGGGTCAACACATCCACG	
*Fxr*	CTGGCATCTATGAACTCAGGC	96
	CCATTCGCGGCTTCTTTGT	
*Cyp7a1*	GGGATTGCTGTGGTAGTGAGC	100
	GGTATGGAATCAACCCGTTGTC	
*β-actin*	F: CCTTCCTTCTTGGGTATGGA	81
	R: ACGGATGTCAACGTCACACT	

**Table A2:** Specific information of primary antibodies used in this study.

Primary Antibody	Species	Dilution Ratio	Catalog Number	Company
FXR	Rabbit	1:1000	A24015	Abclonal
SRB1	Rabbit	1:2000	ET1602-32	Huabio
StAR	Rabbit	1:1000	8449T	CST
CYP11A1	Rabbit	1:2000	HA722814	Huabio
HSD3B1	Rabbit	1:5000	D222511	Sangon Biotech
CYP19A1	Rabbit	1:1000	R23529	Zenbio
CYP7A1	Rabbit	1:3000	18054-1-AP	Proteintech
β-ACTIN	Rabbit	1:20,000	HA722023	Huabio
GAPDH	Rabbit	1:10,000	ET1601-4	Huabio

**Table A3:** Differentially abundant hepatic bile acids identified by targeted metabolomic analysis.

NO.	Bile Acids	Group (ng/g)		VIP	*p*- Value	Fold Change
		CON	ER			
1	CDCA	643.42 ± 19.09	284.34 ± 21.05	1.39	<0.001	0.44
2	UDCA	556.86 ± 52.67	229.91 ± 52.90	1.28	0.012	0.41
3	TCA-3S	335.50 ± 18.26	138.61 ± 16.44	1.38	0.001	0.41
4	3-oxo-CA	0.22 ± 0.11	0.57 ± 0.10	1.25	0.022	2.62
5	UCA	3.85 ± 0.84	322.99 ± 90.95	1.25	0.025	83.97
6	7-KDCA	0.00 ± 0.00	287.57 ± 35.23	1.38	0.015	Inf
7	DCA-3-O-S	0.00 ±0.00	0.93 ± 0.11	1.39	0.001	Inf
8	7,12-DKLCA	0.00 ±0.00	69.44 ± 14.93	1.32	0.010	Inf
9	CDCA-3S	8.55 ± 4.34	110.18 ± 24.33	1.26	0.015	12.89
10	3β-CA	10.31 ± 2.18	46.10 ± 8.38	1.29	0.014	4.47
11	CA	1094.37 ± 210.69	11,293.06 ± 1846.26	1.34	0.005	10.32
12	DCA	47.77 ± 7.99	157.46 ± 20.99	1.32	0.008	3.30
13	HCA	58.39 ± 11.43	122.94 ± 12.88	1.25	0.020	2.11
14	Tω-MCA	16,855.75 ± 2552.68	57,120.07 ± 1895.07	1.40	<0.001	3.39
15	THCA	187.10 ± 33.05	768.91 ± 84.34	1.34	0.003	4.11
16	Tβ-MCA	111.66 ± 9.83	522.00 ± 49.82	1.36	0.001	4.67

**Table A4:** Differentially abundant serum bile acids identified by targeted metabolomic analysis.

NO.	Bile Acids	Group (ng/mL)		VIP	*p*- Value	Fold Change
		CON	ER			
1	3-oxo-DCA	4.72 ± 0.71	34.56 ± 1.79	1.29	<0.001	7.32
2	CDCA	0.00 ± 0.00	274.11 ± 27.22	1.27	0.010	Inf
3	LCA-3S	0.00 ± 0.00	0.95 ± 0.24	1.14	0.017	Inf
4	3-oxo-CA	0.00 ± 0.00	2.82 ± 0.75	1.15	0.020	Inf
5	6,7-DKLCA	0.00 ± 0.00	3.34 ± 0.81	1.17	0.015	Inf
6	7,12-DKLCA	0.00 ± 0.00	24.62 ± 4.09	1.23	0.027	Inf
7	UCA	0.00 ± 0.00	136.14 ± 21.90	1.23	0.025	Inf
8	alloLCA	0.00 ± 0.00	28.16 ± 3.23	1.26	0.001	Inf
9	3β-CA	0.00 ± 0.00	24.43 ± 4.55	1.21	0.006	Inf
10	βGCA	0.00 ± 0.00	7.37 ± 1.57	1.19	0.043	Inf
11	MDCA	4.47 ± 4.47	42.29 ± 9.40	1.15	0.022	9.45
12	ω-MCA	224.39 ± 80.77	14,416.96 ± 3636.38	1.16	0.018	64.25
13	DCA	98.35 ± 46.46	571.65 ± 22.25	1.27	0.001	5.81
14	HDCA	11.49 ± 7.90	270.45 ± 34.40	1.25	0.002	23.54
15	23-DCA	434.42 ± 26.23	1057.35 ± 138.26	1.19	0.011	2.43
16	LCA	3.51 ± 1.82	11.81 ± 1.23	1.17	0.019	3.36
17	CA	139.66 ± 101.26	3688.47 ± 879.11	1.17	0.016	26.41
18	UDCA	8.92 ± 5.51	200.48 ± 20.04	1.27	0.001	22.48
19	7-KDCA	14.05 ± 11.87	2344.15 ± 163.99	1.28	0.005	166.84
20	3β-DCA	12.61 ± 4.75	68.40 ± 5.26	1.27	0.001	5.42
21	isoCDCA	39.38 ± 9.33	157.43 ± 28.07	1.17	0.016	4.00
